# Predictive Testing for Tumor Predisposition Syndromes in Pediatric Relatives: An Asian Experience

**DOI:** 10.3389/fped.2020.568528

**Published:** 2020-10-30

**Authors:** Jianbang Chiang, Jeanette Yuen, Tarryn Shaw, Hui Xuan Goh, Shao-Tzu Li, Eliza Courtney, Joanne Ngeow

**Affiliations:** ^1^Cancer Genetics Service, Division of Medical Oncology, National Cancer Center Singapore, Singapore, Singapore; ^2^Lee Kong Chian School of Medicine, Nanyang Technological University, Singapore, Singapore

**Keywords:** predictive testing, cascade, hereditary cancer, pediatric, Asia

## Abstract

Approximately 10% of pediatric cancer patients possess germline pathogenic/likely pathogenic variants (PV/LPV) in known tumor predisposition genes. Predictive testing is the optimal approach to identify asymptomatic at-risk relatives to guide gene-directed surveillance for early cancer detection and/or risk-reducing strategies. However, the uptake rate for predictive testing remains low in Asian countries. We aim to evaluate the uptake rate of predictive testing in a pediatric population (aged under 21-years-old) in a multi-ethnic Asian cancer center. Our retrospective analysis included families with PV/LPVs identified in genes associated with pediatric tumor predisposition. Of the 83 pediatric first-degree relatives (FDRs) from 49 unrelated families, 20 FDRs (24.1%) originating from 13 families (26.6%) underwent predictive testing. Genes tested in pediatric FDRs were *APC, RB1, SBDS, SDHA, SDHB, SDHD*, and *TP53*. All pediatric FDRs of probands with PV/LPVs in *RB1* and biallelic PVs in *SBDS* underwent predictive testing, while <45% of pediatric FDRs had predictive testing for familial PV/LPVs identified in the *APC, SDHA, SDHB, SDHD*, and *TP53* genes. Amongst the 13 families who underwent pre-test counseling, 80% of pediatric FDRs in these families proceeded with predictive testing. Malay pediatric FDRs and siblings of probands were more likely to undergo predictive testing. We conclude that the predictive testing rate in pediatric FDRs is higher than that of adult FDRs in Asia, but still below the global average. We postulate factors that may influence predictive testing uptake in pediatric FDRs includes a lack of genetics awareness, concerns regarding insurance, and genetic discrimination.

## Introduction

Approximately 10% of pediatric cancer patients have a hereditary monogenic cause (1–3), although the true prevalence is likely higher due to unknown syndromes or the limitations of current DNA sequencing methods (4). Tumor predisposition syndromes, such as familial adenomatous polyposis (FAP) and hereditary retinoblastoma (RB) can affect children, afflicting individuals as young as 10 years old with adenomatous polyposis (5) and new-born infants with retinoblastoma (6), respectively. The majority of pediatric tumor predisposition syndromes follow an autosomal dominant inheritance pattern; first-degree relatives (FDRs) of a proband have a 50% chance of inheriting the familial pathogenic/likely pathogenic variant (PV/LPV). Genetic testing allows for the identification of a PV/LPV in probands, which then sets in motion predictive testing within the family. High rates of predictive testing are beneficial to both the proband's family and the healthcare system. Predictive testing can reduce public healthcare costs and increase efficiency compared to genetic testing of symptomatic probands (7, 8). The uptake rate of predictive testing has a direct impact on cost-effectiveness of genetic testing programs (7, 8) and overall health outcomes (9). On a larger scale, this likely translates to greater cost-savings for the healthcare system as such a model of preventive medicine aims to reduce the burden of cancer-related morbidity and mortality (8–10). Predictive testing is important for pediatric-onset conditions as it provides potentially actionable information for screening asymptomatic children. Correspondingly, family members who test negative can avoid unnecessary screening, medical interventions, and associated costs. Increased genetic awareness and accessibility has improved the uptake of germline genetic testing globally (11–14), providing probands and parents/guardians the opportunity to ascertain if the personal or family history of cancer is hereditary. Results from genetic testing can empower decisions that promote early cancer detection through options, such as intensified surveillance and/or risk-reducing strategies to mitigate cancer risk (15–20).

Rates of predictive testing vary globally, however uptake is consistently lower in Asian countries (7, 21–24). The uptake of predictive testing is dependent on several factors, such as the cost of testing with limited coverage by healthcare institutions, genetic discrimination and reliance on probands to disclose the identification of a hereditary condition among family members (25). Cost remains a significant barrier despite reduction over the past decade with the advent of next-generation sequencing (7, 25). The cost of genetic testing in most parts of Asia is paid out-of-pocket, with minimal government or insurance subsidy. Secondly, there is a lack of legislation to protect against genetic discrimination, including health insurance. This plays an even larger role in the pediatric population who may find that they are unable to obtain insurance coverage due to their underlying hereditary condition. Thirdly, the dissemination of genetic testing results relies solely on the proband (or parents/guardians in cases where the proband is a minor). This hampers predictive testing uptake as proband -initiated disclosure is often complicated by several factors on an individual, familial and cultural basis (21, 23, 26–28). In most parts of Asia, the diagnosis of cancer is stigmatized and rarely discussed among family members, creating another barrier to uptake of genetic testing (28). The proband or parents/guardians may choose not to share genetic results due to distant family relations, fear of discrimination, backlash from family members, as well as perceived burden knowing one has an increased risk of cancer (22, 23).

The Cancer Genetics Service (CGS) at the National Cancer Center Singapore (NCCS) follows the American Academy of Pediatrics (AAP) and the American College of Medical Genetics and Genomics (ACMG) guidelines (4, 29, 30) and recommends predictive testing for pediatric patients only in childhood-onset conditions. To our knowledge, there has been no published literature on predictive testing in pediatric FDRs to date. This study evaluated the uptake rate of predictive testing for pediatric tumor predisposition syndromes in minor FDRs in an Asian cancer center and explores potential factors that affect the uptake rate.

## Methods

Probands who were seen at the CGS at NCCS from March 2014 to December 2019 and had an identified PV/LPV following genetic testing were recruited. Probands were included up until December 2019 to allow for a follow-up period for any delay in predictive testing decisions. Demographic, clinical data, and pedigrees of probands and their pediatric FDRs were extracted from the CGS database (REDCap Software, version 6.10.3, 2017, Vanderbilt University). The database and pedigrees were reviewed by two independent study personnel. Pediatric FDRs of probands who did not attend the CGS clinic were assumed to have declined predictive testing, in tandem with their parents/guardians' decision. Demographic and clinical data for untested FDRs were obtained from pedigrees provided by probands. Financial status of untested pediatric FDRs were assumed to be similar to that of the proband as they are likely to reside in the same household.

Only probands with a PV/LPV in genes associated with pediatric-onset tumor predisposition syndromes were included in the study, in line with AAP and ACMG guidelines. These included *AIP, ALK, APC, ATM, AXIN2, BAP1, BLM, BMPR1A, CDC73, CDKN1C, CEBPA, DICER1, DIS3L2, EPCAM, EXT1, EXT2, FH, GATA2, GPC3, HRAS, LZTR1, MAX, MEN1, MLH1, MSH2, MSH6, NBN, NF1, NF2, PHOX2B, PMS2, PRKAR1A, PTCH1, PTEN, RB1, RECQL4, REST, RET, RUNX1, SDHA, SDHAF2, SDHB, SDHC, SDHD, SMAD4, SMARCA4, SMARCB1, SMARCE1, STK11, SUFU, TERC, TERT, TMEM127, TP53, TSC1, TSC2, VHL, WRN*, and *WT1*. The mismatch repair genes and the *SBDS* gene were tested only if FDRs were at risk of constitutional mismatch repair deficiency (CMMRD) and Shwachman-Diamond syndrome, respectively. Probands were excluded from the study if they were not Singapore residents as their family members were unlikely to be living in Singapore and would have been unable to attend the CGS for predictive testing. A minor, by Singapore law, is defined as an individual under age 21 years and hence the pediatric population is defined as individuals below 21 years old. Written informed consent and assent for medical record research was obtained from all probands and tested FDRs at the point of genetic testing. The study was approved by the Singhealth Centralized Institutional Review Board (CIRB number 2010/826/B).

Genetic counseling services at NCCS are provided by medical oncologists with specialization in genetics and/or Master's trained genetic counselors. A shared decision-making approach for pre-test genetic counseling is adopted in the CGS (31). Following the identification of a PV/LPV in a proband, family notification letters were provided to assist the proband/family members with dissemination of the result. Family members who were keen to undergo genetic testing were referred to the CGS where an appointment for pre-test genetic counseling was scheduled to facilitate predictive testing.

Tested and untested pediatric FDRs were compared for potential prognostic factors of predictive testing uptake. Two-tailed chi-square test and independent samples *t*-test were performed for categorical and normally distributed continuous variables, respectively. For categorical variables with a 2 × 2 distribution, a two-tailed Fisher's exact test was used when the expected count was below 5. Statistical significance was set at P < 0.05. All statistical analyses were performed using IBM SPSS version 25.

## Results

**Figure 1:**
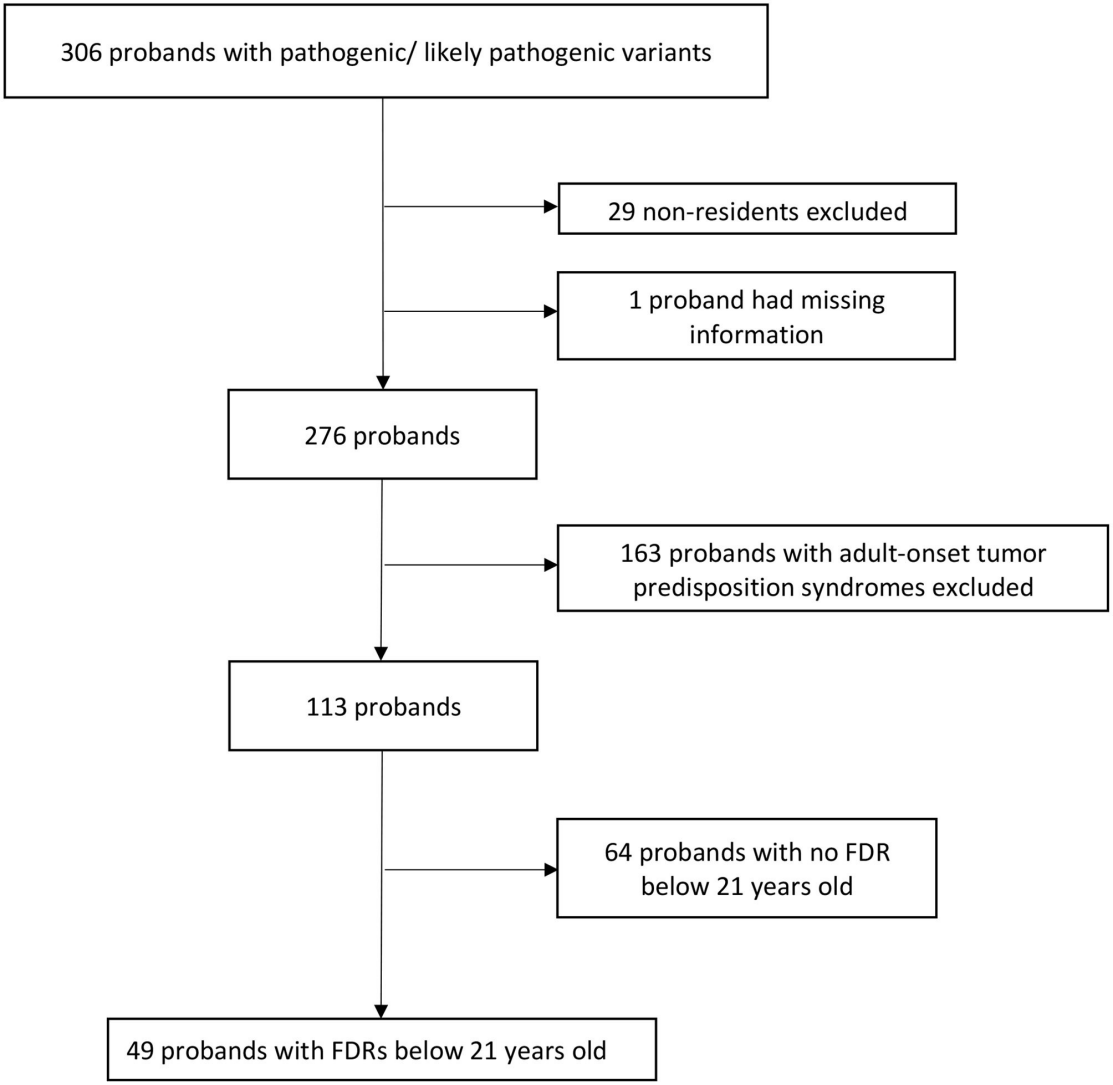
Exclusion criteria of study. FDRs, first-degree relatives.

Overall, 306 probands who underwent genetic testing between March 2014 and December 2019 were found to have PV/LPVs in known tumor predisposition genes. After excluding 29 non-residents, one proband with missing information, 163 probands with adult-onset tumor predisposition syndromes and 64 probands with no FDRs below 21 years old (Figure 1), there were 83 pediatric FDRs from 49 unrelated probands. A total of 20 pediatric FDRs (24.1%), originating from 13 families (26.6%), underwent predictive testing (Table 1).

**Table 1:** Proportion of FDRs below 21 years old and families who had predictive testing.

**FDRs below 21 years old**			**Source families**		
**Total**	**Tested**	**Not tested**	**Total**	**Tested**	**Not tested**
	**(%)**	**(%)**		**(%)**	**(%)**
83	20 (24.1)	63 (75.9)	49	13 (26.6)	36 (73.5)

Demographic and clinical information of the 49 probands whom carried an identified PV/LPV in a pediatric-onset tumor predisposition gene and the 20 pediatric FDRs who had predictive testing are shown in Table 2. The mean age of the probands and pediatric FDRs were 35.0 and 11.3 years, respectively. The majority of probands were female (63.3%), Chinese (77.6%), and had a personal history of cancer (91.8%). In comparison, the pediatric FDRs who underwent testing were similar in terms of gender (female; 50.0%) and ethnicity (Chinese; 70.0%). The ethnic distribution in probands and pediatric FDRs is representative of the Singaporean population (32). Most of the pediatric FDRs did not have a personal history of cancer (90.0%). The need for financial assistance was similar between probands and pediatric FDRs, at 32.7 and 35.0%, respectively. Overall, the familial PV/LPV was detected in 11/20 (55.0%) of tested pediatric FDRs.

**Table 2:** Demographic and clinical factors of probands and tested pediatric FDR.

	**Probands (*n* = 49)**	**Pediatric FDRs (*n* = 20)**
**AGE**		
Mean (range)	35.0 (1–57)	11.3 (3–20)
**SEX**		
Male (%)	18 (36.7)	10 (50.0)
Female (%)	31 (63.3)	10 (50.0)
**RACE**		
Chinese (%)	38 (77.6)	14 (70.0)
Malay (%)	6 (12.2)	6 (30.0)
Indian (%)	2 (4.1)	0 (0.0)
Others (%)	3 (6.1)	0 (0.0)
**PERSONAL HISTORY OF CANCER**		
Yes (%)	45 (91.8)	2 (10.0)
No (%)	4 (8.2)	18 (90.0)
**FINANCIAL ASSISTANCE**		
Yes (%)	16 (32.7)	7 (35.0)
No (%)	33 (67.3)	13 (65.0)
**GENETIC RESULT**		
Positive (%)	49 (100.0)	11 (55.0)
Negative (%)	0 (0.0)	9 (45.0)

Pediatric FDRs underwent predictive testing for familial PV/LPVs identified in the following genes: *APC, RB1, SBDS, SDHA, SDHB, SDHD*, and *TP53* (Table 3). Among six unrelated probands with identified *APC* PV/LPVs, there were 18 pediatric FDRs. Eight pediatric FDRs (44.4%) from three families (50.0%) underwent predictive testing for the familial *APC* variant. Two pediatric FDRs from one family had genetic testing for familial PVs in *APC* and *MUTYH* as there were two PVs found in the proband. There were two unrelated probands with identified *RB1* PV/LPVs with three pediatric FDRs from both families. All three pediatric FDRs from both families (100.0%) had predictive testing. One family had a PV in both *RB1* and *TP53*. One proband with biallelic *SBDS* PVs had one pediatric FDR who underwent predictive testing (100.0%). Of 16 pediatric FDRs from 12 families with *SDHx* PV/LPVs, seven pediatric FDRs (43.8%) from six families (50.0%) underwent predictive testing. Out of nine pediatric FDRs from seven families with *TP53* PV/LPVs, three FDRs (33.3%) from two families (28.6%) had predictive testing for the familial variant. More than half of the eligible pediatric FDRs did not proceed with predictive testing for familial PV/LPVs identified in *APC, SDHx*, and *TP53*. Among the 13 families that presented for predictive testing, 20/25 (80.0%) pediatric FDRs underwent predictive testing.

**Table 3:** Proportion of FDRs who underwent predictive testing by gene.

**Gene**	**Genetic Condition**	**FDRs below 21 years old**			**Source families**		
		**Eligible for testing**	**Tested (%)**	**Not tested (%)**	**Eligible for testing**	**Tested (%)**	**Not tested (%)**
*APC*	Familial adenomatous polyposis	18	8 (44.4)	10 (55.6)	6	3 (50.0)	3 (50.0)
RB1^*^	Hereditary retinoblastoma	3	3 (100.0)	0 (0.0)	2	2 (100.0)	0 (0.0)
*SBDS*	Shwachman-Diamond syndrome	1	1 (100.0)	0 (0.0)	1	1 (100.0)	0 (0.0)
*SDHx (SDHA, SDHB, SDHD)*	Hereditary paraganglioma-pheochromocytoma syndrome	16	7 (43.8)	9 (56.2)	12	6 (50.0)	6 (50.0)
TP53^*^	Li-Fraumeni Syndrome	9	3 (33.3)	6 (66.7)	7	2 (28.6)	5 (71.4)

* *Two FDRs within one family underwent predictive testing for pathogenic variants in TP53 and RB1, both found in the proband*.

We identified two factors that shows significant association with the uptake of predictive testing in pediatric FDRs—ethnicity and relationship to proband (Table 4). Malay pediatric FDRs were more likely to undergo predictive testing as compared to other ethnic groups (66.7 vs. 23.0%, *p* = 0.005). In addition, pediatric siblings of probands were more likely to undergo predictive testing compared to children of probands (53.3 vs. 17.6%, *p* = 0.003). We examined other potential factors that may affect the uptake of predictive testing, although we did not find any significant associations with gender, age of FDR, age of parents/guardians, or socioeconomic status.

**Table 4:** Factors associated with uptake of predictive testing in FDRs below 21 years old.

	**Tested**	**Not tested**	***P*-value**
**AGE OF FDR**			
Mean (range)	11.3 (3–20)	9.1 (0–20)	0.141
**SEX**			
Male (%)	10 (25.0)	30 (75.0)	0.853
Female (%)	10 (23.3)	33 (76.7)	
**RACE**			
Chinese (%)	14 (23.0)	47 (77.0)	**0.006**^a^
Malay (%)	6 (66.7)	3 (33.3)	
Indian (%)	0 (0.0)	8 (100.0)	
Others (%)	0 (0.0)	5 (100.0)	
**MEAN AGE OF PARENTS/GUARDIANS**			
Mean (range)	40.3 (32–56)	42.4 (28–57)	0.251^b^
**RELATIONSHIP TO PROBAND**			
Child (%)	12 (17.6)	56 (82.4)	**0.003**
Sibling (%)	8 (53.3)	7 (46.6)	
**FINANCIAL ASSISTANCE**			
Yes (%)	7 (25.9)	20 (74.1)	0.787
No (%)	13 (23.2)	43 (76.8)	

a *Fisher's Exact test*.

b *Independent sample t-test*.

*Chi-square test was used, unless otherwise specified*.

*Bold values indicate statistical significance p < 0.05*.

## Discussion

This study reports the predictive testing uptake rate in pediatric FDRs of probands with PV/LPVs in pediatric tumor predisposition genes. Concurrently, it provides insight into the uptake of commonly tested genes among pediatric FDRs of Asian families.

We observed a 24% uptake rate of predictive testing for tumor predisposition syndromes in the Singaporean pediatric population, almost double the predictive testing rate of 13% in Singaporean adults (25). The lack of predictive testing data for pediatric-onset tumor predisposition syndromes meant that there were no available data for comparison. We postulate that the low predictive testing rate in our Asian pediatric population may be due to a combination of factors relating to poor genetics knowledge and awareness, concerns regarding insurance and genetic discrimination, and Asian familial culture.

There is a general lack of understanding of the clinical utility of genetic testing in Singapore (23). This could explain the poor uptake of predictive testing amongst potential pediatric *APC, SDHA, SDHB, SDHD, a*nd *TP53* PV/LPV carriers, who may be at increased risk for a range of different cancer types from a young age. Our data demonstrates that pediatric FDRs are significantly less likely to undergo predictive testing if the proband is the parent. We hypothesize that parents/guardians may want to minimize invasive procedures, such as blood tests, which cause the child unnecessary worry. They may also be concerned that knowledge of a hereditary condition may result in stigma from the family/community and have an impact on the child's psychological well-being, which in turn could impact schooling, social interaction, and self-esteem. Parents/guardians may also have difficulty broaching the subject of hereditary conditions and explaining the risk to their children, possibly stemming from guilt of passing it on to the next generation (33). Furthermore, parents/guardians may project assumptions onto the child, which may make for inaccurate assessments of the child's ability to understand and/or cope with the implications of undergoing predictive testing. Such assumptions may be overly paternalistic, as there are varying levels of cognitive maturity in the two decades spanning the pediatric age group, where adolescents are known to be capable of independent thoughts that may be distinct from their parents/guardians. Parents/guardians may worry that the child is not mature enough to understand the impact of genetic information (33, 34). Often in Asia, clinical consultations with pediatric FDRs comprises of an extended discussion with the parents/guardians, with minimal interaction with the child. The CGS at NCCS actively overcomes this by involving the child in an age-appropriate way throughout the pre-test counseling process with developmentally-appropriate explanations, child-friendly assent forms, and engaging them in the final decision-making, where appropriate. Unfortunately, we are aware of instances where information has been intentionally withheld by parents/guardians to protect their at-risk child(ren) from the knowledge of an increased risk of cancer, despite the provision of family communication strategies between parents/guardians and child.

From an ethical point of view, the subject of predictive testing in pediatric FDRs is keenly debated (35–38). Advocates highlight the actionability of identifying pediatric PV/LPV carriers to guide early screening to detect cancer at an earlier and more manageable stage or risk-reducing interventions, with the aim of decreasing mortality. This is especially observed in pediatric patients with familial adenomatous polyposis (FAP), where colorectal adenomatous polyposis and cancer can develop at a young age (39, 40). Genetic testing for the purpose of enhancing medical monitoring, prophylaxis or treatment in pediatric FDRs may be in the best interest of the child in such conditions (41). Detractors cite the right to autonomy and self-determination of the child as a reason to defer germline testing until they are able to comprehend the spectrum of benefits and limitations (42), especially as there are often reproductive and insurance implications following germline genetic testing. The best interests of the child must be respected at all times and healthcare providers need to balance the autonomy of the child and medical need for genetic testing carefully. The balance might come from testing children only when cancer risk begins in childhood and where there are evidence-based interventions to mitigate such risks.

Interestingly, our service reports a predictive testing rate in pediatric FDRs that is nearly double that of adult FDRs (25). Previous studies of adults in Singapore who underwent genetic testing found several barriers to disclosure of results by the proband, including cost, concerns regarding insurance, potential genetic discrimination, as well as perceived burden of genetic results (27). This barrier of proband-mediated disclosure is not unique to Asia, with literature demonstrating similar challenges in other countries (43, 44). In the case of a pediatric FDR, proband-mediated disclosure is not a relevant factor as parents/guardians are often involved in the entire genetic counseling process.

Medical decision making in Asia usually includes significant input from the family, especially in the Malay community (22, 28, 45). In Asian culture, the concept of illness is familial, rather than individual, and involvement of the family provides hope, support, and strength (46). This pattern of familial decision-making can be seen as entire families often come together for testing if they choose to do so and vice versa (25). In our dataset, Malay pediatric FDRs, whom traditionally apply a familial decision-making approach (28), are more likely to undergo predictive testing than other ethnic groups. We observed that predictive testing tends to happen in clusters within families which suggests the strong influence of the family in decision-making for genetic testing. Further research on family-based genetic counseling should be considered in Asia.

Based on our study, *RB1* was the most common gene tested when predictive testing was offered to pediatric FDRs. Even though all *SBDS* pediatric FDR had predictive testing, this should be interpreted with caution as it is based on a single proband with biallelic *SBDS* PVs with one pediatric FDR. Hereditary retinoblastoma is a disease of childhood and curative intervention can be performed if detected early. The *RB1* gene is highly penetrant with most carriers presenting with retinoblastoma before age five (6). Parents/guardians may thus be more likely to opt for early testing to improve detection and prospects of cure.

Complete data, with minimal missing information, is a strength of this study. Though numbers are small, our study addresses a gap in the literature by looking at the issue of predictive testing uptake in pediatric FDRs and sets a benchmark for comparison with future studies. Further studies with larger datasets would be beneficial for comparison. Our study did not explore the reasons for or against predictive testing in children, such as the breakdown of age, education, and socioeconomic status. Future qualitative studies are required to understand the concerns and needs of pediatric FDRs and their parents/guardians (47, 48). Additionally, pedigree and family information was dependent on proband's recall which may be subject to recall bias. Our study has limited access to FDRs who may have undergone predictive testing via other services, which may have led to an underestimation of predictive testing uptake rates. Nevertheless, this is unlikely to be a significant number as our center has funding assistance for testing and the majority of predictive testing is done at the same center as the proband.

## Conclusion

This study addresses a question that has not been reviewed in literature, by demonstrating that a quarter of pediatric FDRs undergo predictive testing for childhood-onset tumor predisposition syndromes in Asia. While the rate is higher than that observed in adult FDRs in Singapore, it is still below global predictive testing rates. Factors, such as ethnicity and relationship-to-proband are positive predictors for the uptake of predictive testing amongst pediatric FDRs. Future directions for further exploration include facilitators and barriers to predictive testing unique to a pediatric population, addressing lack of protective legislature especially for health insurance, the effectiveness of family-based genetic counseling in improving pediatric predictive testing uptake, and/or the approach of directly contacting FDRs for predictive testing without proband-mediated dissemination.

## Data Availability Statement

The original contributions presented in the study are included in the article/supplementary materials, further inquiries can be directed to the corresponding author/s.

## Ethics Statement

The studies involving human participants were reviewed and approved by Singhealth Centralized Institutional Review Board (CIRB number 2010/826/B). Written informed consent to participate in this study was provided by the participants' legal guardian/next of kin.

## Author Contributions

JC and JN: conception and design and provision of study materials or patients. JC: administrative support, collection and assembly of data, and data analysis and interpretation. JC, JY, TS, HG, S-TL, EC, and JN: manuscript writing and final approval of manuscript. All authors contributed to the article and approved the submitted version.

## Conflict of Interest

The authors declare that the research was conducted in the absence of any commercial or financial relationships that could be construed as a potential conflict of interest.
